# Case Report: False vertebral artery stenosis: an rare phenomenon

**DOI:** 10.3389/fsurg.2025.1598842

**Published:** 2025-10-07

**Authors:** Yan Nao, Yang Niao, Sun Dong, Mei Bin

**Affiliations:** 1 Department of Neurology, Zhongnan Hospital of Wuhan University, Wuhan, Hubei, China; 2 Hubei Provincial Clinical Research Center for Dementia and Cognitive Impairment, Zhongnan Hospital of Wuhan University, Wuhan, Hubei, China; 3 Department of Internal Medicine, Wuhan University of Science & Technology, Hanyang Hospital, Wuhan, Hubei, China

**Keywords:** trans-radial cerebral angiography, vertebral artery stenosis, simmon-2, catheter, loop, case report

## Abstract

**Introduction:**

Trans-radial access (TRA) has gained widespread adoption in neurointerventional procedures, particularly for cerebral angiography. The success of trans-radial cerebral angiography crucially relies on the proper formation of the simmons-2 catheter loop to navigate the aortic arch and selectively catheterize the cerebral vessels. However, the potential of simmons-2 catheter loop configuration to induce angle change of the vasculature was not previously recognized in our institutional practice.

**Methods:**

We present a rare cerebrovascular imaging phenomenon identified during diagnostic and therapeutic neurointerventional procedures in a 60-year-old male patient. Selective cerebral angiography performed via right radial access demonstrated severe ostial stenosis of the right vertebral artery. Thus, endovascular stent placement was scheduled to address the right vertebral artery stenosis via trans-radial approach. Surprisingly, intraoperative vertebral artery angiography via 6F guiding catheter demonstrated complete resolution of the preoperative stenosis. We conducted a comparative analysis of subclavian artery and brachiocephalic trunk morphology using pre- and post-loop formation angiographic imaging.

**Results:**

Angiographic analysis revealed that the simmons-2 catheter loop configuration resulted in a significant alteration of the angle between the horizontal subclavian artery and the descending brachiocephalic trunk, producing vertebral artery traction and apparent stenosis.

**Discussion:**

The discrepancy arose from a previously unrecognized phenomenon: simmons-2 catheter loop configuration altered the angulation between the vessels, resulting in artefactual vertebral artery ostium stenosis during initial angiography. This case reveals previously unrecognized anatomic-vascular interactions influencing for trans-radial neurointerventional procedures. This report carries significant implications for procedural techniques and image interpretation in neurointerventional practice.

## Introduction

Trans-radial cerebral angiography has gained increasing recognition among neuro-interventionalists as a preferred approach, offering superior patient comfort, reduced access-site complications, and comparable efficacy to traditional transfemoral method. Proper simmons-2 loop formation is critical to trans-radial cerebral angiography success (1–3). Standard cerebral angiography sequence at our institution: left vertebral artery → left common carotid artery → right common carotid artery → right vertebral artery (4). In our actual clinical practice, right vertebral artery angiography is performed via two technical approaches: the loop-release method and direct subclavian cannulation approach. The first approach involves controlled release of the simmons-2 catheter loop in the aortic arch with subsequent retrograde positioning at the vertebral origin. Alternatively, operators may employ direct subclavian cannulation using the simmons-2 catheter loop, analogous to internal carotid artery selection technique. However, comparative analysis of vertebral arteriograms obtained through these two approaches demonstrates that superselective subclavian arteriograms eliminates interference from carotid systems, thereby yielding clearer visualization.

Although the simmons-2 catheter loop may inherently applies mechanical tension to the subclavian vessel, prior cases showed no clinically significant difference between loop-release and direct subclavian approaches. Notably, our literature review revealed no prior reported case of simmons-2 catheter-induced vascular stenosis secondary to mechanical traction. Previous research has primarily focused on technical safety, patient comfort, and the learning curve associated with the trans-radial approach vs. the transfemoral approach (5). This report reveals a previously overlooked biomechanical interaction, providing novel insights into catheter-vessel interactions during neurointerventional procedures. The Institutional Review Board of Zhongnan Hospital, Wuhan University (Approval#2024215K) Written informed consent was obtained from the individual for the publication of any potentially identifiable images or data included in this article.

## The process of the two operations

**Figure 1:**
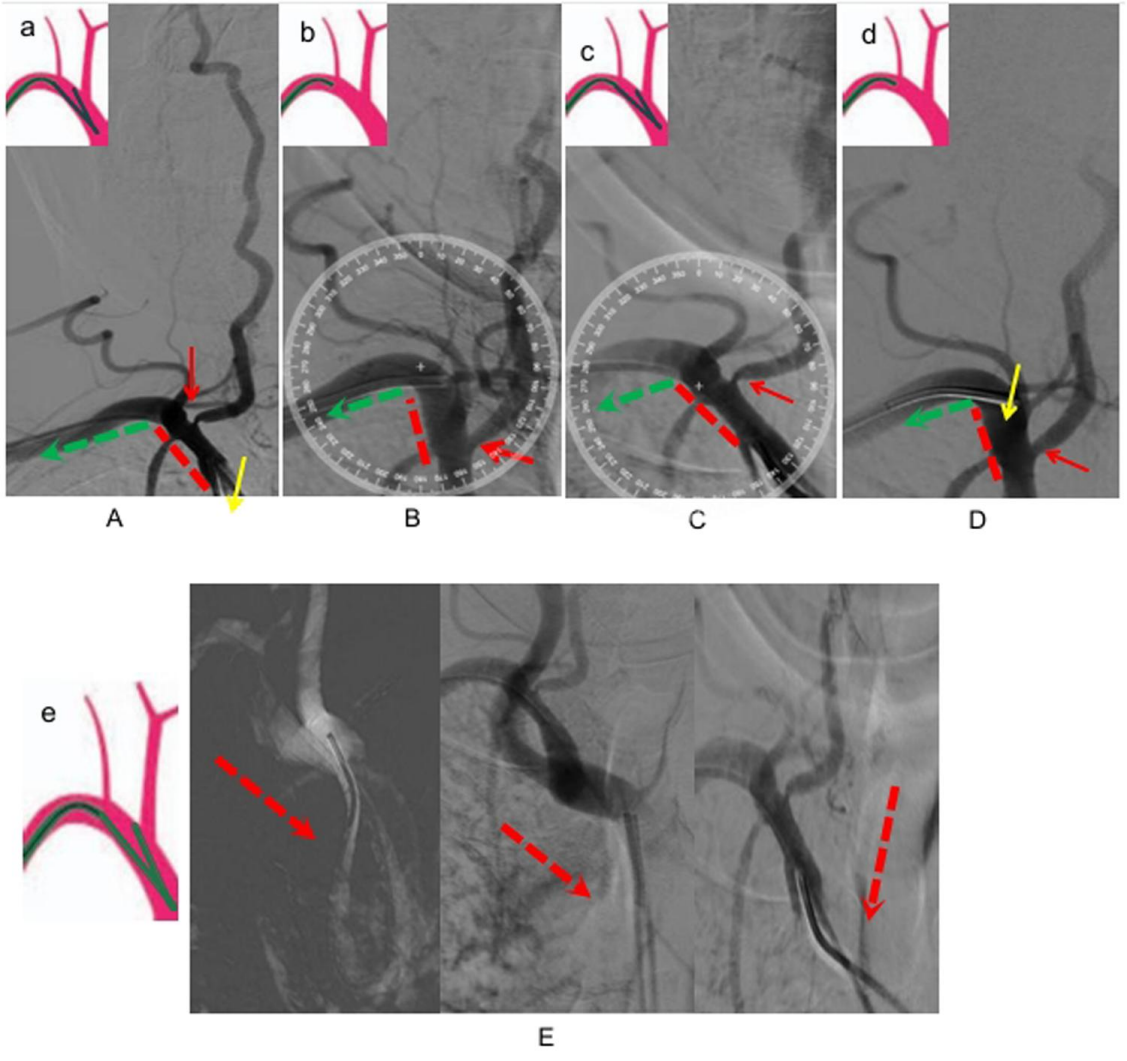
**(A)** Right vertebral arteriography by a loop formation with a simmon-2 catheter, indicating severe stenosis of the vertebral artery, a: schematic diagram of simmon-2 catheter loop formation and subclavian arteriography was selected. **(B)** Right vertebral arteriography by a 6F catheter, three days later. **(C)** The 6F catheter was replaced with the same simmon-2 catheter, right vertebral arteriography via a forming simmon-2 catheter during the operation. **(D)** The forming catheter was unravalled and then retreats to the right vertebral artery initiation at the same angle for angiography, d: Schematic diagram of Simmon-2 catheter retracting to the beginning of the vertebral artery after unraveling the forming loop. **(E)** The simmon-2 catheter's loop configuration, incompletely visulalized in **(A,C)**. e: Schematic diagram of simmon-2 catheter loop.

A 60-year-old male was admitted to a local hospital one week prior following the acute onset of right upper extremity weakness and sensory impairment. Brain MRI demonstrated an acute cerebral infarction in the left frontoparietal region, and MRA revealed: stenosis of the left MCA (middle cerebral artery) M1 segment, bilateral ACA (anterior cerebral artery) narrowing with luminal stenosis. For definitive vascular evaluation, the patient was admitted to our department. Right trans-radial cerebral angiography revealed severe ostial stenosis at the origin of the right vertebral artery during the procedure (Figure 1A). We adopted the second approach at that time, where right vertebral artery angiography was achieved through super-selective catheterization using a simmons-2 catheter loop in the subclavian artery. After optimal medical therapy (OMT) and multidisciplinary risk assessment, right vertebral artery ostial stenting was planned to perform using a right trans-radial approach. Notably, intraprocedural vertebral artery angiography using a 6F guiding catheter did not reveal the previously detected significant vertebral artery ostium stenosis (Figure 1B).

What could account for the rapid disappearance of significant vertebral-ostial stenosis over such a brief interval? Naturally, our primary consideration was suboptimal angiographic projection. Thus, we re-evaluated the index procedural angiograms from three days prior and performed repeat angiography with meticulously replicated projection angles. However, even after meticulous angle adjustment matching the initial angiographic projections, no significant stenosis was observed at the right vertebral artery origin. We conducted a critical methodological comparison of the two angiographic procedures. The initial diagnostic angiogram, conducted three days before the intervention, used a 5F simmons-2 catheter with a standard loop formation technique to selectively engage the right subclavian artery for vertebral artery opacification. For the current therapeutic process, right vertebral artery angiography was performed through targeted ostial cannulation replicating the catheter withdrawal technique following simmons-2 catheter loop reformation—a standard maneuver for proximal vertebral artery access. Are the dynamic changes in vertebral artery stenosis formation and disappearance mechanistically associated with the simmons-2 catheter loop? We reselected the simmons-2 catheter to perform super-selective subclavian angiography after loop formation and reproduced severe stenosis at the origin of the vertebral artery using the same projection angles (Figure 1C). And when we released the simmons-2 catheter loop and withdrew the catheter to perform angiography at the origin of the vertebral artery, the severe stenosis disappeared, consistent with the findings from the 6F catheter angiography (Figure 1D). Therefore, this unexpected phenomenon can be explained by tensile forces from the simmons-2 catheter loop causing the angle change of the vasculature and pseudo-stenosis of the vertebral artery.

## Discussion

TRA cerebral angiography is gradually becoming an alternative to TFA cerebral angiography. In recent years, an increasing number of studies have demonstrated that TRA cerebral angiography is associated with fewer complications, as well as better patient comfort and satisfaction (6). Our center primarily employs trans-radial access (TRA), predominantly via the right radial approach, for cerebral angiography. The simmons-2 catheter loop is essential for achieving rapid and effective vessel selection in trans-radial cerebral angiography (7). Our cerebral angiography protocol follows a systematic left-to-right sequence: left vertebral artery → left carotid artery → right carotid artery → right vertebral artery. Typically, we reconstitute the simmons-2 catheter shape before withdrawing it to position the tip at the vertebral artery origin in the right subclavian artery (Figures 1A,C). However, we sometimes also perform direct vertebral artery angiography by selectively advancing the loop-formed simmons-2 catheter into the right subclavian artery(Figures 1B,D). The structural morphology of simmon-2 catheter's loop is as shown in Figure 1E, which is also the part not depicted in Figures 1A,C. Moreover, prior reports overlook that simmons-2 catheter loop formation and subclavian selection induce substantial vascular tension, risking deformation of the subclavian-brachiocephalic angle.

In this particular case, angiographic morphology of the subclavian artery and brachiocephalic trunk was evaluated by comparing vascular configurations during formed and non-formed catheter loop states. The subclavian-brachiocephalic trunk angle changed significantly from ∼80° to ∼120°.As shown from Figures 1B,C, we have measured the angle between subclavian artery and brachiocephalic trunk. During vertebral artery angiography with a simmons-2 catheter loop in the brachiocephalic trunk artery (as shown in Figure 1E), the angle between the subclavian artery and the brachiocephalic trunk is approximately 120 degrees, resulting in significant stenosis of the vertebral artery (as shown in Figures 1A,C). However, when we released the simmons-2 catheter loop and withdrew the catheter to the subclavian artery,the angle between the subclavian artery and the brachiocephalic trunk was approximately 80 degrees (as shown in Figures 1B,D), During the two procedures for this case, we used the same simmon- 2 catheter and the same glidewire, without employing a stiff glidewire that could alter the catheter's configuration and lead to different outcomes. Both operations were performed with identical materials and techniques, consistent with our standard practice. Therefore, we believe that the simmons-2 catheter loop exerts traction force on the brachiocephalic trunk in this case, which leads to changes in the angle between the subclavian artery and the brachiocephalic artery, consequently resulting in pseudostenosis of the vertebral artery.

This unexpected phenomenon uncovers a previously undocumented mechanical interplay between catheter and vasculature during manipulation. This case demonstrates that catheter-induced tension can create artifactual vascular morphology. We present this previously unreported phenomenon to advance understanding of trans-radial cerebral angiography.

## Conclusion

The change of the subclavian-brachiocephalic complex angle may resulted in pseudostenosis of the vertebral artery. This previously unrecognized phenomenon offers critical implications for biomechanical interaction and image interpretation in trans-radial neuro-angiography.

## Data Availability

The datasets presented in this article are not readily available because of ethical and privacy restrictions. Requests to access the datasets should be directed to the corresponding authors.
